# Feasibility and acceptability of an adapted WHO alcohol brief intervention: Pilot of a three-armed randomized trial in Sri Lanka^☆^

**DOI:** 10.1016/j.puhip.2025.100704

**Published:** 2025-12-17

**Authors:** Dewasmika Ariyasinghe, Sally Carter, Cathy Banwell, Buddhima Lokuge, Thilini Rajapakse, Grace Joshy, Kamalini Lokuge

**Affiliations:** a National Centre for Epidemiology and Population Health, School of Health and Medicine, Australian National University, ACT, 2601, Australia; b Hunter New England Local Health District, Newcastle, NSW, Australia; c University of Newcastle, Newcastle, NSW, Australia; d Department of Psychiatry, Faculty of Medicine, University of Peradeniya, Sri Lanka; e Teaching Hospital Peradeniya, Sri Lanka

**Keywords:** Alcohol brief interventions, (BI), Risky drinking, (RD), Family burden, AUDIT score, Cultural adaptation, Sri Lanka

## Abstract

**Background:**

Risky drinking (RD) is a major health hazard in Sri Lanka. Alcohol brief intervention (BI) has been proven effective in minimizing RD but has not been utilised in Sri Lanka. We therefore aimed to adapt the WHO alcohol BI and targeted educational material to Sri Lanka, assess their feasibility and acceptability and evaluate appropriateness of methodology and measures for a future RCT.

**Study design:**

A three-arm parallel-group pilot RCT.

**Methods:**

The BI was adapted based on expert feedback. The study included male inpatients (with AUDIT-C screening score ≥5) of a tertiary hospital. The three study arms were: adapted brief intervention (ABI), education about unit of alcohol (UOA), and feedback on screening results (FOA). Trained research assistants (RAs) screened and implemented the interventions. We report on follow-up rates (feasibility), participant and RA feedback (acceptability), recruitment efficiency and data quality (methodological appropriateness), and appropriateness of outcome measures.

**Results:**

The ABI included a structured training manual for implementers, an alcohol information leaflet, and a personal information sheet. Patient follow-up rates were 69 %, 40 % and 71 % for FOA, UOA and ABI arms respectively. Family member recruitment was 31 %. Patient and RA feedback for ABI was overwhelmingly positive. Many patients were abstinent at baseline (37.5 %) and follow-up (75.9 %), mainly due to health concerns. FMQ revealed high ‘total family burden’. Patients struggled with TLFB recall. High childhood adversity prevalence (95.7 %) and low alcohol knowledge were observed.

**Conclusions:**

The ABI demonstrated high acceptability among patients and RAs. All three interventions could be trialled in a future RCT. All measures except TLFB proved appropriate. Our innovative approach of evaluating outcomes from family members' perspectives proved feasible and valuable. The inpatient setting was not appropriate, rather a setting where patients continue their day-to-day activities, including usual drinking, should be considered in a future RCT.

1 **What this study adds**

• This study developed the first culturally adapted intervention for addressing alcohol misuse specifically for Sri Lanka, a setting with very high levels of risky drinking and alcohol related harms.
• To our best knowledge, this is the first study to demonstrate the feasibility and acceptability of using family members' perspectives as an outcome measure alongside patient perspectives in the same study, representing an important outcome independent of individual patient benefits.
• Inpatient settings may be inappropriate for testing the effectiveness of alcohol brief interventions as many patients reduce or stop drinking due to their health conditions and hospitalisation.

2 **Implications for policy and practice**

This pilot study provides important guidance for the effective implementation of future RCTs assessing ABI, and other interventions to address alcohol misuse. This guidance will ensure that such RCTs are able to provide valid answers to research objectives that are critical to addressing a significant health problem in Sri Lanka and globally.

## 3 Introduction

Alcohol is ranked as the most harmful substance of abuse when combining harms to self and others, and the third most harmful substance to users [1]. Users experience higher rates of malignancies, infections, metabolic syndrome, injuries, cognitive deficits, and poverty. Non-users are also affected by injuries, crimes, family adversities, and poverty to mention a few. Most alcohol-related harms are attributed to heavy episodic consumption, not to alcohol dependence [2].

Sri Lanka faces significant challenges with risky drinking (RD) - drinking at levels or in patterns that increase the risk of alcohol-related harms [3]. Heavy episodic drinking affects 9 % of those =>15 years and 32 % of drinkers, despite approximately half the population being lifetime abstainers. Eighty-five percent of drinkers consume spirits [4]. Local research reveals the impact of alcohol misuse in depression among drinkers’ spouses [5], domestic violence [6], suicide [7,8], economic costs [9], accidents [10,11], and child abuse [12].

Despite this high prevalence of RD and adverse consequences on the individual and the society, Sri Lanka lacks an effective behavioural intervention for opportunistic use with risky drinkers that is feasible for widespread implementation.

### 3.1 What are brief interventions?

The WHO endorses brief interventions (BIs) for harmful and hazardous alcohol use. BIs use non-confrontational conversations to motivate individuals to contemplate changing their drinking and related behaviours. BIs emphasize patient autonomy, patient-centred collaboration, and treating behaviour change as a process, not an event [13].

### 3.2 What is the importance of this pilot RCT?

Client-informed decision making facilitates change [14]. Conversely, paternalistic approaches and abstinence demand—both common in Sri Lanka—deter behavioural change [15], highlighting the need for BI-centred approaches.

Opportunistic interventions— delivered to individuals not seeking alcohol-related help—enhance efficiency [16] but require numerous trained individuals. Given the limited availability of specialists, training non-specialists to deliver these interventions is essential if such interventions are to be accessible to all those in need. Several studies demonstrate that non-health staff can effectively deliver interventions after proper training to address RD in a range of settings, which also promotes better client engagement due to reduced stigma [17,18].

Cultural adaptation is necessary given variation in drinking habits and cultural values, and it improves acceptability and effectiveness [19,20]. While unstructured unadapted BIs were previously used in Sri Lanka [21], culturally adapted structured interventions are needed, especially when implemented by non-specialist or non-health staff.

Previous research on BIs reveals several deficiencies: demonstrating efficacy rather than effectiveness, susceptibility to the Hawthorn effect^1^ [22], over-reliance on self-reports, inadequately evaluated content [23], and limited evidence from lower-income settings [24,25]. Furthermore, studies typically measure effectiveness solely through consumption changes, overlooking improvements in family environment—important aspects in harm reduction. Addressing these requires widening inclusion criteria, using non-specialist interventionists [26], incorporating family perspectives as outcome measures, and developing structured, culturally adapted manuals.

WHO's BI manual encourages use of decisional balancing—exploring the pros and cons of continuing versus stopping or reducing alcohol consumption— in client-centred discussions, an important construct in the transtheoretical model [13,27]. However, eliciting this information can be time-consuming, and non-specialists may struggle with raising such sensitive issues [17]. BIs also take about 25 min to administer [28]. In resource-limited settings like Sri Lanka, this could significantly impact efficient implementation. We therefore included two less resource-intensive interventions with hierarchical structure: 'Feedback on AUDIT-C' (FOA) and 'Unit of Alcohol education' (UOA). FOA was the foundational component and was included within UOA, while UOA was a subcomponent of the ABI (further details provided in methods). If proven effective in future RCTs, these less resource intense interventions would be particularly valuable.

### 3.3 Adverse childhood experiences and knowledge of alcohol related harms as potential effect modifiers

Adverse childhood experiences increase the risk of alcohol use disorders through neurobiological [29] and permanent neuro-hormonal changes [30] and may affect intervention outcomes [31]. Poor knowledge of alcohol harms and low-risk drinking (LRD) predicts RD [20], and knowledge reduces long-term harms [32,33]. However, evidence on how prior knowledge affects BI effectiveness is lacking [34]. Determining whether these factors modify intervention effects is important for targeting and adaptation.

### 3.4 Study aims

1) adapt WHO alcohol BI for delivery by non-specialists in Sri Lanka.
2) assess feasibility and acceptability of a) the adapted BI (ABI), b) targeted educational material (unit of alcohol education- UOA) and c) giving feedback on screening (FOA).
3) Evaluate methodology for a future RCT by (a) assessing the appropriateness of the study design and setting, (b) determine the appropriateness of outcome measures in both patient and family member perspectives, and (c) explore the feasibility of measuring adverse childhood experiences and alcohol knowledge to assess effect modification.

## 4 Methods

### 4.1 Phase 1: adapting the BI

Cultural sensitivity is a key principle in public health [19]. The Primary Investigator (PI) adapted the BI using Resnicow's cultural adaptation principles, focusing on surface and deep structures [19], while preserving the OARS framework (Open-ended questions, Affirmations, Reflections, Summaries) of WHO [13], with enhanced contextualization [35]. Five psychiatric specialists experienced in alcohol-related issues (two local, one Australia-based, and two former local specialists now working in Australia) provided feedback through multiple refinement cycles.

### 4.2 Phase 2: pilot RCT

#### 4.2.1 Context

The study was conducted at Teaching Hospital Peradeniya (THP), central Sri Lanka, which provides free health care to patients from diverse socioeconomic backgrounds.

#### 4.2.2 Participants and eligibility

Due to marked gender differences in alcohol use disorders (AUD) prevalence in Sri Lanka (males 5.9 %, females 0.7 %) [4], only males were recruited.

Inclusion criteria.

• Adult male inpatients from medical, neurology, surgical, orthopaedic, and toxicology wards
• Risky drinkers - AUDIT-C score ≥5 [13] at screening- Validated Sinhala AUDIT-C [36] was updated per recent guidelines [14].

Exclusion criteria.

• Serious illness
• Current AUD therapy or electing to access specialised care
• Inability to communicate in the language the research assistants were conversant with (Sinhala)

#### 4.2.3 Study design and procedure

A three-arm parallel group pilot study design included ABI, UOA and FOA arms (section 2.3). All eligible male inpatients received study information; consenting patients completed screening. Eligible patients consented to participation and family member contact (spouse, adult sibling, parent, or adult child). Family member consent was also sought. Participants and family members were reimbursed Rs. 2500 (∼7.25 USD) per assessment for transport costs and their time to participate [37].

**Fig. 1:**
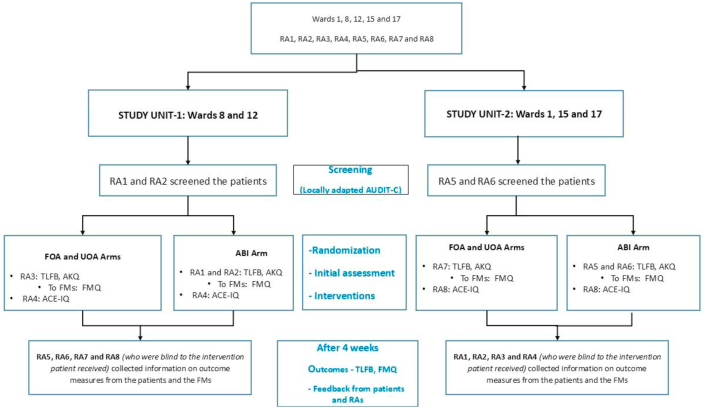
Allocation of research assistants, measures used and randomization in the two study units (RA-research assistant, FMs-family members, FOA- Feedback on AUDIT-C, UOA-unit of alcohol, ABI- adapted brief intervention, AUDIT-C: Alcohol Use Disorders Identification Test-C, TLFB- Timeline Follow Back, AKQ-Alcohol Knowledge Questionnaire, FMQ-Family Member questionnaire, ACE-IQ-Adverse Childhood Experiences- International Questionnaire).

The five wards were divided by proximity into two study units, each with four research assistants (Fig. 1). Screening RAs recorded eligible patients, then different RAs randomized those who consented using an ABC, BCA, CAB pattern in each unit. During real-world implementation, the same practitioner would likely complete screening and intervention delivery, utilizing any rapport developed during screening. However, only RAs allocated to the ABI arm were trained in ABI to prevent contamination—ensuring non-ABI RAs couldn't use ABI skills in their respective arms. Consequently, this approach, where the same RA delivers screening and intervention was only feasible for the ABI arm (Fig. 1).

The four-week follow-up assessment used RA swapping between units to facilitate unbiased outcome assessment. Initial assessment blinding was impossible due to the nature of the intervention.

#### 4.2.4 Sample size

Data collection continued until each arm reached 15 patients.

### 4.3 Interventions

#### 4.3.1 FOA

RAs informed the patient about his AUDIT-C score meaning i.e. their consumption is likely to have caused problems already or is likely to cause problems in future. The alcohol leaflet, prepared for ABI, was then given to them. This involved the provision of one-way information, with no interactive discussion with the patient.

#### 4.3.2 UOA

In addition to all components of FOA, these patients received brief education from the RA on UOA and LRD (i.e.: maximum 2 units/day, activities to avoid after drinking, risks of bingeing 5 ≥ units/occasion). There was no interactive discussion.

#### 4.3.3 ABI

In addition to all components of UOA, RAs used the ABI manual and ‘patient information sheet (PIS)’ (section 3.1) to engage the patient in a discussion. This included a two-week text reminder as well.

### 4.4 Outcome measures

The study measured three main outcomes: (i) Feasibility and acceptability of the ABI, UOA and FOA, (ii) feasibility of the methodology, appropriateness of measures of intervention outcomes and (iii) feasibility of measuring effect modifiers. See columns 2 and 3 of Table 1.

**Table 1:** Summary of objectives, methods and results.

Objectives	Construct	Methods	Results
**Assess the feasibility and acceptability of**			
***a) the adapted BI (ABI)***	Duration	Direct observation	**15**–**30 min**
	Retention	Follow-up attendance	**Patient 70.6 % (12/15), FM-85.7 % (6/7)**
	Acceptability	***Patient feedback questionnaire****:* Modified from a previously used questionnaire [38], this assessed satisfaction with ABI content, setting, duration, RA qualities and perceived effectiveness, on a 5-point scale (Completely disagree, disagree, undecided, agree, agree completely), with two open-ended questions for suggestions and comments.	− 10 patients completed the feedback questionnaire - -**Feedback was overwhelmingly positive.** - All patients identified the ABI **manual-guided discussion as the most helpful componen**t over the leaflet and text reminding of their plan - Patients’ suggestions for improvement - community implementation **(‘we have so many in our neighbourhood, who would benefit from this’**), more frequent interactions **(‘we wish we had several meetings’).** Several had commented **‘I often think of this [the ABI] when I think of a drink’**.
		***RA feedback questionnaire:*** Modified from an intervention facilitator feedback tool [39], the questionnaire used the same 5-point scale, to assess RA perceptions of patient reception, training adequacy, delivery confidence, intervention duration and ABI components, with two open-ended questions for suggestions and comments. The feedback was given anonymously.	– **Feedback was overwhelmingly positive in** all components assessed with recommendations for a separate space to minimize disturbance by ward activities - Further, the **information gathered from patient information sheet (PIS) had greatly facilitated the initial engagement with the patient.**
***b) targeted educational material (UOA)***	Duration	Direct observation	**∼5 min**
	Retention	Follow-up attendance	**Patient 40 % (6/15), FM – 50 % (1/2)**
	Acceptability	Not assessed	–
***c) Feedback on AUDIT-C (FOA).***	Duration	Direct observation	**∼3 min**
	Retention	Follow-up attendance	**Patient -68.8 % (11/16), FM – 66.7 % (4/6)**
	Acceptability	Not assessed directly	Follow-up rates are encouraging
**Explore the appropriateness of**			
***a) Methodology***	Patient recruitment rate	Screening records	25.3 (48/190)
	FM recruitment rate	Consent records	45.4 % (15/33)
	Patient follow-up rate	Attendance tracking	60 % (29/48)
	FM follow-up rate	Attendance tracking	73 % (11/15)
	Setting	- Time taken for recruitment of patients - Abstinence rates - Family member recruitment rate	- Data collection was completed in nine days − 38 % (17/45) were abstinent at the initial assessment − 76 % (22/29) were abstinent at follow-up - It was hard to meet the family members due to **incompatibility of hospital visiting hours and RA working hours**
***b) Outcome measures***	Time taken to complete and comprehensibility	***Timeline Follow Back (TLFB):*** Retrospectively assesses alcohol consumption over periods up to 12 months [40]. This study used TLFB to estimate consumption in the 28 days preceding each assessment.	RAs feedback - **Daily drinkers** completed assessment quickly (∼2 min) with accurate recall, while **non-daily drinkers** struggled significantly (up to 10 min) with poor consumption recall.
		***Family Member Questionnaire (FMQ):*** Interviewer-administered 30-item assessment measuring social, emotional, physical, and financial impacts of patient drinking on family members, coping strategies and support, using a 4-point scale (0 = 'Never', 1 = 'Once or twice', 2 = 'Sometimes', 3 = 'Often'). Intervention effectiveness determined by reduced 'Total family burden' combining impact domains, symptoms, emotional coping, and tolerant inactive coping [41] (Appendix 1). Questionnaire was translated into Sinhala, back-translated, and corrected.	RAs feedback -Family members understood the questionnaire well. Administration took 10–15 min. **Several expressed relief for getting an opportunity to discuss their concerns** and requested healthcare referrals. ‘Total family burden’ appeared to be high, but no statistical analysis was done.
**c) Evaluate the feasibility of measuring adverse childhood events and alcohol knowledge as potential effect modifiers.**		***Alcohol Knowledge Questionnaire (AKQ):*** Developed by the PI the questionnaire assessed patients' basic alcohol knowledge, including 13 alcohol-related harms from the WHO ABI manual [13] and awareness of UOA and LRD. RAs administered the questionnaire	- **Well comprehended**; administration took ∼10 min. - Only 23.4 % and 12.8 % reported awareness of UOA and LRD respectively, though accuracy of their knowledge wasn't verified. While 90 % recognized accident risk and 62 % knew about weight gain, knowledge of other alcohol harms was below 38 % each.
		***Adverse Childhood Experiences: International Questionnaire (ACE-IQ):*** The translated and validated version of the WHO questionnaire [42] was used.	- **Well-comprehended** - **Took ∼20**–**30 min to complete**. − **95.7 % (45/47) reported at least one childhood adversity** (Appendix 2).

### 4.5 Selection, training, allocation and supervision of research assistants

Eight RAs were selected through interviews, focusing especially on their communication skills. None had substantial training/work experience as health staff; some had done short counselling internships. The ten-day training by the PI included lectures, role-plays and hands-on sessions covering study purpose, basic alcohol knowledge, UOA and LRD concepts, alcohol-related societal harms and mental illness, research ethics, consent, and administering the study measures. Post-training, four RAs with better communication skills—a quality sought in previous non-specialist BI studies [18] - received ABI training and were assigned to ABI delivery, two RAs to FOA and UOA to minimize contamination, and two RAs to adverse childhood questionnaire administration. RAs handled screening, consent, randomization, data collection, and intervention delivery. The PI made regular visits to the wards to ensure RAs followed the study protocol and to help with any difficulties they faced. The PI also held weekly meetings with the RAs to discuss issues, and RAs could contact the PI by phone anytime.

### 4.6 Analysis

Descriptive analysis used Stata/MP 17.0. Qualitative feedback was thematically analysed to improve future ABI development. Outcome quantification identified factors requiring attention in future RCTs.

## 5 Results

### 5.1 Phase-1-final adapted brief intervention

The final ABI included an illustrated leaflet with information on alcohol and LRD, patient information sheet – PIS, and a manual. Initiating discussion on alcohol and acquiring information to tailor-make the discussion is time consuming and challenging, even for a health care professional [43]. PIS - a questionnaire to gather information on the patient's income, who he associates with when drinking, reasons/occasions he drinks, life goals, any alcohol-related problems he has faced if any, and whether he had received any information on alcohol prior - was developed to address this issue. The manual was to enable RAs to tailor discussions based on responses to PIS.

### 5.2 Phase-2

#### 5.2.1 Patient characteristics

Patient characteristics and outcome measures for each arm are detailed in column four of Table-1. The column also includes family member follow-up rates. Risky drinkers (AUDIT-C ≥5) were significantly younger than non-risky drinkers (45.47 ± 14.03 vs. 53.98 ± 19.04 years, p = 0.005, 95 % CI: 2.59–14.42). The study sample (n = 48) had mean values of age 45.5 ± 14.0 years, education 9.0 ± 3.7 years, and AUDIT-C score of 7.4 ± 1.9. Mean patient ages across FOA, UOA, and ABI arms were 47.3, 45.6, and 43.8 years respectively. Mean years of education were 9.7, 8.4, and 9.0 years respectively, while mean AUDIT-C scores were 13.6, 12.9, and 17.3 in the same order.

#### 5.2.2 Methodology

**Figure-2:**
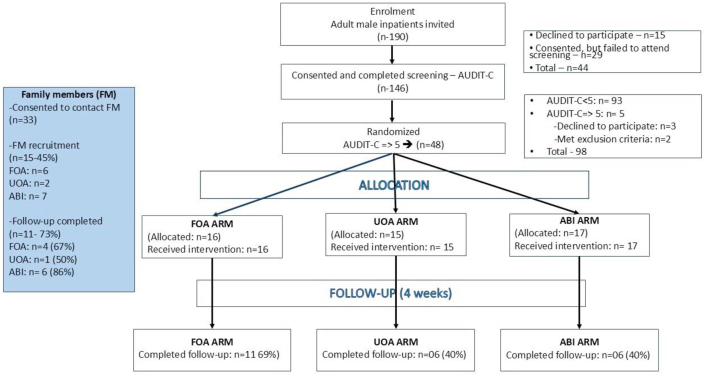
The CONSORT flow-chart of patient selection, randomization, and follow-up and information on family members (FOA- Feedback on AUDIT-C, UOA- Unit of alcohol, ABI- adapted brief intervention, TLFB-Timeline Follow-back, FMQ- Family member questionnaire.

Data collection was completed within nine days (Fig. 2). Notably, 38 % (17/45) of participants at initial assessment and 76 % (22/29) at follow-up were abstinent, with at least 50 % and 32 % of them respectively attributing it to ongoing illness. Other reasons cited were ‘no money’, ‘did not have parties during the 28 days’.

The PIS revealed primary drinking motivations: socialization/happiness [n = 34], coping with negative emotions [n = 23], and habit [n = 9]), along with common misconceptions about alcohol (that it eliminates negative emotions, improves eating/sleeping, and cannot be refused).

#### 5.2.3 Outcome measures

Results are summarized in column four of Table 1. Overall follow-up rates were 60 % for patients and 73 % for family members. Childhood adversity details appear in appendix 2.

## 6 Discussion

To our knowledge, this study represents the first adaptation and piloting of an ABI for risky drinkers in Sri Lanka, using Resnicow's principles [19] and WHO guidelines [13]. Resnicow describes surface structure as matching interventions to population characteristics for cultural 'fit', and deep structure as addressing cultural, social, historical, and psychological forces influencing health behaviours. We incorporated both: surface structure through non-stigmatizing language and culturally modified dialogues drawing on the PI's extensive psychiatric experience with AUD patients, plus UOA illustrations for clarity; and deep structure by addressing alcohol myths, demographic factors and stigma revealed by the PIS. This adaptation maintains impact [19] while preserving the WHO's OARS framework (Open-ended questions, Affirmations, Reflections, Summaries) and elements including personalized feedback, advice, alternative options, moderate strategies, 'change talk', enhancing self-efficacy and role-playing to build patient assertiveness [13].

The three components of the ABI— leaflet, PIS, and the manual—work synergistically. Information from PIS provides a starting point to raise concerns, at which many interventionists are anxious in the first place [44], and also gives tailor-make direction for the ABI. Positive feedback from patients and RAs, combined with reasonable follow-up rates, confirmed acceptability and feasibility of ABI. Poor knowledge of alcohol harms and LRD emphasizes the importance of the leaflet in addressing these educational needs, both within interventions and for broader population health initiatives [32]. The PIS efficiently captured drinking motivations and misconceptions, potentially reducing intervention time compared to sessions without this preparatory tool. Its comprehensibility, combined with Sri Lanka's high literacy rate [45], suggests patients could complete it while waiting to see practitioner. Overall implementation time matched international studies [28].

**Fig. 3:**
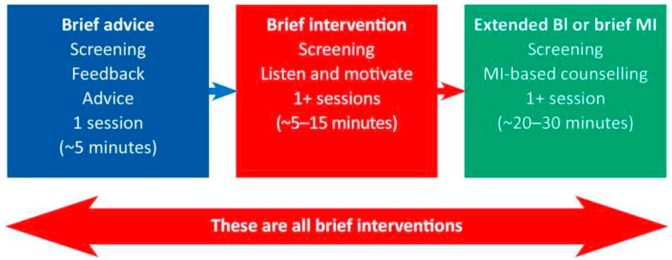
**A continuum of brief interventions** Source: WHO alcohol brief intervention training manual for primary care. BI-Brief intervention, MI- Motivational interviewing.

We included UOA and FOA arms - which could be considered as BIs (Fig. 3) - because they require less time and resources than ABI. Despite UOA's lowest follow-up rate, its brief nature suits the local context and warrants inclusion in future RCTs. Despite being the least intense, follow-up rates of FOA for both patients and family members were encouraging. To maximize benefit, the PIS should also be given to patients in these arms in future powered RCT to encourage reflection—helping those in precontemplation reconsider their drinking through consciousness raising and self-re-evaluation [27].

Recruitment feasibility was demonstrated by a 25 % eligibility rate and nine-day completion. However, high baseline and follow-up abstinence rates, with a considerable proportion citing illness-related factors as the reason, suggest future effectiveness studies should use primary healthcare settings where patients maintain their usual drinking patterns.

Regarding outcome measures, non-daily drinkers struggled with 4-week TLFB recall, indicating need for an alternative patient-outcome measure. The FMQ effectively captured family impacts and revealed valuable insights, with some requesting further care referrals. While initially burdensome for services, this likely improves overall outcomes - the impact of RD on family member wellbeing and vice versa is clearly documented [46] and is also an important factor described in the transtheoretical model of behaviour change [27]. This aspect could be addressed in future outpatient-based RCTs, where family members typically accompany patients.

The AKQ and ACE-IQ were acceptable, though ACE-IQ was time-consuming. Poor alcohol knowledge and very high ACE prevalence may reflect broader public health concerns, highlighting their importance as potential effect modifiers in future trials and need for large-scale prevalence studies.

Risky drinkers were significantly younger than the non-risky drinkers, consistent with local [47] and international research [2], suggesting age as a useful screening factor.

### 6.1 Limitations

Several constraints affected this study. We omitted polysubstance use and psychiatric comorbidity assessments to avoid overburdening hospitalized participants, though these are potential confounders. We didn't evaluate RA micro-skills (listening, communication), which may influence outcomes more than content itself [23]. Family member recruitment was limited by scheduling difficulties, but conducting a future RCT in a primary health care facility will address this, since the patient is usually accompanied by a family member to the appointment. Further, patient and RA feedback was not collected for the UOA and FOA arms, limiting direct assessment of their feasibility and acceptability. Feedback questionnaires for these arms will be added in the future RCT. Follow-up rates were moderate and need improvement strategies, like more reminder phone calls. Further, dropouts may sometimes occur when participants improve and do not see need for follow-up until relapse—a rarely researched perhaps a common clinical observation. Follow-up at least over the phone could be added in the methodology to investigate this aspect. Interventions were conducted only in Sinhala; adaptations will be needed for other Sri Lankan communities.

### 6.2 Strengths of the study

Our study attempted to address several limitations in BI research [23]. Given the heterogeneity in duration, number of sessions, content and intensity—ranging from single sessions of ∼5–30 min to multiple sessions, and from screening with feedback to screening and motivational counselling (Fig. 3) [13], it is hard to compare ABI effectiveness studies. When setting, training level of practitioner and severity of alcohol consumption are added, comparing BI studies becomes nearly impossible. Poor intervention documentation further hinders reproducibility [48,49]. Our study clearly documented intervention time, content for each arm, structured ABI delivery, and RA selection, qualifications, and training. Selection of RAs with reasonably good communication skills at the beginning ensured that each intervention is delivered optimally. Furthermore, the current pilot study aimed to measure effectiveness, not efficacy, through broader inclusion criteria, and using non-specialists which will increase generalizability [26].

Including family perspectives was a major innovation rarely implemented in alcohol intervention research, despite documented negative impact of drinkers on family members and vice versa, leading to unhealthy outcomes in both [41,46]. This dual-perspective methodology addresses two other significant limitations in traditional alcohol research: participant under-reporting, and failure to capture harm reduction in family dynamics [3], that may occur independently of changes in drinking - a meaningful outcome in itself. The study demonstrated feasibility of measuring family member perspective as an outcome measure, thus enabling holistic assessment of intervention impacts beyond mere reduction of alcohol consumption.

We developed the ABI for the Sri Lankan context - designed for implementation by non-specialist personnel. Our RAs had no health sector work experience yet reported confidence and passion after 10-day training. This is quite promising considering issues raised in previous research - lack of knowledge and low confidence in persons delivering BI, unavailability of materials, along with concerns about raising alcohol-related issues with clients [44]. Overwhelmingly positive patient feedback, rating discussions as most helpful, supports non-specialist training and intervention feasibility. Patient comments such as 'So many in our neighbourhood would benefit,' 'We wish we had several meetings,' and 'I often think of this when I think of a drink', further support this.

Incorporating different BI intensities in the same RCT proved feasible with clearly documented procedures. Conducting all interventions in the same setting is likely to eliminate contextual effects. These are initial steps in trying to untangle the confusion in terminology and reduce heterogeneity of BIs, thereby making it easier to make comparisons and to decide which BI is effective and resource efficient in what setting.

### 6.3 Conclusions

This pilot demonstrated ABI feasibility and acceptability among Sri Lankan male inpatients and their family members. Administering ABI by non-specialists with good communication skills is also feasible and acceptable. Using two less intense brief interventions -UOA and FOA-in the same study was also feasible. All outcome measures except TLFB were appropriate-the FMQ proved particularly valuable for capturing overlooked family perspectives. Alarming levels of poor knowledge of alcohol-related harms, UOA, and LRD, combined with very high levels of adverse childhood experiences are genuine public health concerns; their inclusion as potential effect modifiers in future ABI trials is feasible and essential. However, the inpatient setting proved sub-optimal for evaluating intervention effectiveness due to illness-induced abstinence and difficulty in meeting family members. A future RCT should target primary care settings where patients continue typical day-to-day activities, including their usual drinking patterns, and where family member contact is easier, to better detect intervention effects.

## Ethics statement

The study was approved by the Ethics Review Committee of the Faculty of Medicine, University of Peradeniya, Sri Lanka (2022/EC/56) and the Human Ethics Committee of the Australian National University, Australia (2022/779). The trial was registered at the Sri Lanka Trials Registry (SLCTR/2022/027).

## Author contribution

DA designed the study and CB, BL, TR, GJ and KL suggested improvements. DA drafted the manuscript and CB, BL, TR, GJ, SC and KL revised and made improvements.

## Data availability statement

Data will be available on request from the authors.

## Funding

The study was funded by the Romaine Rutnam scholarship of the Australian National University, Canberra, Australia. The tuition fee of the principal author was paid by 'Full Time Higher Degree Research Scholarship' awarded by the Australian National University, Canberra, Australia

## Declaration of Competing Interest

The principal author and TR are specialists in psychiatry working at Teaching Hospital Peradeniya, Sri Lanka, where this study was carried out, may have had contact with some participants/family members recruited for the study. Other five authors declare no conflict of interest.

## Footnotes

1 Describes the behavioural change due to an awareness of being observed or positive response to the intervention.
