# Supplementary material for: Feasibility and acceptability of an adapted WHO alcohol brief intervention: Pilot of a three-armed randomized trial in Sri Lanka
Source: Public Health Pract (Oxf). 2025 Dec 17;11:100704. doi: 10.1016/j.puhip.2025.100704 (PMC12796105; doi:10.1016/j.puhip.2025.100704)
Supplement: Multimedia component 1 [file mmc1.docx]

|  | **Score**  **Before intervention** | **Score**  **After intervention** |
| --- | --- | --- |
| ***Impact*** | | |
| TOTAL Impact (sum of Q1-6) ↓ |  |  |
| Worrying behaviour (sum of Q1-3) |  |  |
| Active disturbance (sum of Q4-6) |  |  |
| ***Symptoms*** | | |
| TOTAL Symptoms (sum of Q7-12) ↓ |  |  |
| Psychological symptoms (sum of Q7-9) |  |  |
| Physical symptoms (sum of Q10-12) |  |  |
| ***Coping*** | | |
| Engaged coping emotional (sum of Q13-15) ↓ |  |  |
| Engaged coping assertive (sum of Q16-18) |  |  |
| Tolerant inactive coping (sum of Q19-21) ↓ |  |  |
| Withdrawal coping (sum of Q22-24) |  |  |
| ***Support*** | | |
| Helpful informal support (sum of Q25-27) |  |  |
| Helpful formal support (sum of Q28-30) |  |  |
| Unhelpful informal support (sum of Q31-33) |  |  |
| **TOTAL FAMILY BURDEN** | | |
| Sum the 4 shaded boxes above ↓ |  |  |

**Appendix 1: Family Member Questionnaire (FMQ)**

Reference

Orford, J., et al., *Family members of relatives with alcohol, drug and gambling problems: a set of standardized questionnaires for assessing stress, coping and strain.* Addiction, 2005. **100**(11): p. 1611-24.
