# Supplementary material for: Feasibility and acceptability of an adapted WHO alcohol brief intervention: Pilot of a three-armed randomized trial in Sri Lanka
Source: Public Health Pract (Oxf). 2025 Dec 17;11:100704. doi: 10.1016/j.puhip.2025.100704 (PMC12796105; doi:10.1016/j.puhip.2025.100704)
Supplement: Multimedia component 2 [file mmc2.docx]

| **Type of abuse** | **Frequency (%)** | | **Remarks** |
| --- | --- | --- | --- |
| 1. Emotional abuse | 34 (72.3) | | 29 reported that their parents/ guardians were not adequately aware of what they were doing in their free time |
| 1. Physical abuse | 21 (44.7) | | On 14 occasions an object was used |
| 1. Sexual abuse | 9 (19.1) | | 1 instance of attempted rape |
| 1. Violence against household members | 9 (19.1) | |  |
| 1. Living with household members who were substance abusers | 4 (8.5) | |  |
| 1. Living with household members who were mentally ill or suicidal | 2 (4.2) | |  |
| 1. Living with household members who were imprisoned | 0 (0) | |  |
| 1. 8. One or no parents/ parental separation or divorce/ death of a parent/s | 11 (23.4) | | 10 had experienced the death of a parent |
| 1. Emotional neglect | 33 (70.2) | |  |
| 1. Physical neglect | 6 (1.3) | |  |
| 1. Bullying | 9 (19.1) | |  |
| 1. Community violence | 37 (78.7) | | 32 had seen/ heard someone being beaten up  13 had seen/ heard someone being stabbed/shot  19 had seen/ heard someone being threatened with a knife or gun in real life? |
| 1. Collective violence | 7 (1.5) |  | |

**Appendix 2: Adverse childhood experiences of the patients (n=47)**
